# Validity of parent-reported weight and height of preschool children measured at home or estimated without home measurement: a validation study

**DOI:** 10.1186/1471-2431-11-63

**Published:** 2011-07-07

**Authors:** Inge Huybrechts, John H Himes, Charlene Ottevaere, Tineke De Vriendt, Willem De Keyzer, Bianca Cox, Inge Van Trimpont, Dirk De Bacquer, Stefaan De Henauw

**Affiliations:** 1 Department of Public Health, Faculty of Medicine and Health Sciences, Ghent University, Ghent, Belgium; 2 Division of Epidemiology and Community Health University of Minnesota School of Public Health, Minneapolis, USA; 3 Research Foundation Flanders, Brussels, Belgium; 4 Department of Nutrition and dietetics, Faculty of Health Care, University College Ghent, Gent, Belgium; 5 Unit of Epidemiology, Scientific Institute of Public Health, Brussels, Belgium; 6 Centre for Pupils Counselling (CLB), Flemish Community Education, Ghent, Belgium

**Keywords:** BMI, weight, height, validity, children, parent reports

## Abstract

**Background:**

Parental reports are often used in large-scale surveys to assess children's body mass index (BMI). Therefore, it is important to know to what extent these parental reports are valid and whether it makes a difference if the parents measured their children's weight and height at home or whether they simply estimated these values. The aim of this study is to compare the validity of parent-reported height, weight and BMI values of preschool children (3-7 y-old), when measured at home or estimated by parents without actual measurement.

**Methods:**

The subjects were 297 Belgian preschool children (52.9% male). Participation rate was 73%. A questionnaire including questions about height and weight of the children was completed by the parents. Nurses measured height and weight following standardised procedures. International age- and sex-specific BMI cut-off values were employed to determine categories of weight status and obesity.

**Results:**

On the group level, no important differences in accuracy of reported height, weight and BMI were identified between parent-measured or estimated values. However, for all 3 parameters, the correlations between parental reports and nurse measurements were higher in the group of children whose body dimensions were measured by the parents. Sensitivity for underweight and overweight/obesity were respectively 73% and 47% when parents measured their child's height and weight, and 55% and 47% when parents estimated values without measurement. Specificity for underweight and overweight/obesity were respectively 82% and 97% when parents measured the children, and 75% and 93% with parent estimations.

**Conclusions:**

Diagnostic measures were more accurate when parents measured their child's weight and height at home than when those dimensions were based on parental judgements. When parent-reported data on an individual level is used, the accuracy could be improved by encouraging the parents to measure weight and height of their children at home.

## Background

With a growing interest in childhood obesity as a factor in child morbidity and adult diseases,[1] valid measures of childhood weight and height are of interest to many researchers. Because of the logistical difficulties and financial costs involved in directly measuring weight and height of children in a survey, such data are often proxy-reported (e.g. by the parents) [2-6]. Previous studies focusing on the validity of parent-reported weight, height and body mass index (BMI) values in children have shown fairly poor accuracy of parentally reported values for classifying children into BMI categories of underweight, overweight and obesity status [7-9]. From a recent review of the literature, Himes concluded that proxy measures for directly measured BMI, such as self-reports or parental reports of height and weight, are much less preferred and should only be used with caution and awareness of the limitations, biases, and uncertainties of these measures [10]. Nevertheless, because direct measurements of weight and height are costly and time consuming, large surveys in childhood populations are likely to continue to use parent-reported values. A practical solution to improve the validity of these parent reports could be to ask parents to measure the weight and height of their children at home.

To date, there are no studies evaluating the validity of parental measurements of preschool children in comparison with parental estimates without measurements. The aim of the present study was to compare the validity of parent-reported weight and height values of their child after being measured at home with that of parent estimates of height and weight without home measurement. We also compared the corresponding accuracy of the parent reports for classifying children into BMI categories, using international BMI cut-off values for underweight, overweight and obesity.

## Methods

### Study population

Subjects were residents in the region of Ghent, a medium sized city in Belgium. A sample of 3-7 year-old children was recruited using a multistage cluster sampling technique. First, three school committees were randomly selected in the region of Ghent and they all agreed to participate (a school committee manages/governs one or more schools). In total, these three school committees included seven different school residences/locations. All 29 preschool classes of these seven schools were selected as final cluster units. All the children from these 29 selected classes were invited to participate.

### Questionnaire and self-reported anthropometry

Parents were asked in a questionnaire to report the weight and height of their child. In addition, they were asked to report if they actually measured their child's weight and height prior to reporting, or if they estimated the values without measurement. No protocol or instructions were provided for measuring the child at home. Also, information about the child (e.g. gender and age) and his or her parents (e.g. age and parental education levels) was asked.

### Anthropometric measurements

This study was conducted in collaboration with Centers for Pupils Counselling ('Centrum voor Leerlingenbegeleiding' or 'CLB' in Dutch). Preventive health care and routine medical examinations are performed at the CLBs, including weight and height measurements. All the children participating in this study were examined and measured by a CLB nurse (3 different CLB nurses) in a standardized way (according to the protocol 'VWVJ & Vlaamse Groeicurven')[11]. For these measurements, children were only wearing underwear. Weight was recorded to the nearest 0.1 kg, using an electronic weighing scale (Seca 841) and height was measured to the nearest 0.1 cm in standing position, using a rigid stadiometer (Seca 220). The stadiometer was checked for accuracy and the scale was calibrated before examination.

### Procedures

The directors of the schools and the teachers of the classes participating in the study were given detailed information and instructions about the study.

The teachers of the participating classes were asked to distribute the questionnaire among the parents of the children about 14 days before the routine medical examination in the CLB. An informed consent was attached, in which parents were informed and invited to participate in the study, without being aware that validation of anthropometric measurements was part of the study. The completed questionnaires and the signed informed consents were returned to the school in a sealed envelope. Nurses at the CLB-centers were not allowed to open the sealed envelopes to be sure that they were not influenced by the parent-reported weights and heights.

The Ethical Committee of the Ghent University Hospital granted ethical approval for the study.

### Statistical analysis

BMI (kg/m²) was calculated from parent-reported measurements and estimates of heights and weights. Underweight, overweight, and obesity were identified using age- and gender-specific international (International Obesity Task Force (IOTF)) cut-off points [12,13]. These BMI cut-offs were defined so that they correspond to the same locations in the BMI distributions at younger ages as BMI-values of, respectively, 18.5 kg/m^2^, 25 kg/m^2 ^and 30 kg/m^2 ^at age 18. Even though a multistage sampling design was used, mixed models suggested that the clustering effect is only small and could be ignored. When the difference between measured and parent-reported values were examined the likelihood-ratio test showed that neither school nor class had significant random effects (p = 0.180 and p = 0.147 respectively).

Differences in mean parent-reported and CLB measured weight, height and BMI, and between differences in prevalences of underweight, overweight and obesity were assessed using paired t-test and McNemar's test respectively. Limits of agreement were estimated from the SD of differences from the index measurements (mean difference ± 1.96SD), considering the measurements derived from the CLB nurses as index measurements. Intraclass correlation coefficients between measured and reported values were calculated as a measure of overall association.

When identifying underweight, normal weight, overweight and obesity, misclassification was defined as discordance between BMI-categories, determined by parent-reported and parent-measured BMI versus nurse-measured BMI. The weighted kappa statistic was calculated to determine agreement between parent-reported and measured index BMI-status adjusted for chance, using a linear set of weights. Kappa values range between -1.00 (perfect disagreement) and 1.00 (perfect agreement), with a value of zero suggesting no agreement beyond chance alone. Kappa values less than 0.20 are often considered as "poor" agreement, between 0.21 and 0.40 as "fair" agreement, between 0.41 and 0.60 as "moderate" agreement, between 0.61 and 0.80 as "good" agreement, and between 0.81 and 1.00 as "excellent" agreement [14].

Sensitivity was defined as the probability that a child, categorized in a certain BMI-category (e.g. overweight), based on the measured index BMI, was also categorized in that BMI-category when using parent reports (true positive rate). Specificity was defined as the probability that a child, assigned as not having a certain BMI-status (e.g. overweight), when using measured index BMI, was not assigned to that same BMI-category when using the parent-reported data (true negative rate).

Differences in bias of parent reports between the different reporting methods used by the parents (e.g. height & weight both estimated versus height and weight both measured at home) were studied by comparing the percentage of subjects for whom the difference between reported and measured BMI values was > 1 kg/m^2 ^or < -1 kg/m^2^, according to the reporting type. Fisher's exact test was used to test for statistical significance.

The Statistical Package for the Social Sciences (SPSS) for Windows Version 15 was used for data management and all statistical analyses. Unless reported differently, a P-value of 0.05 (two-sided) was used as the threshold for statistical significance.

## Results

A sample of 474 preschool children were officially registered in the 29 sampled classes. Of those, 26 children could not be included because of absences; questionnaires were returned for 329 children (73%). Complete data were available for 297 children. Comparison of the body measurements of the 297 included children with the children whose parents did not return a questionnaire (n = 32) showed that the children included in the study were slightly taller than those not included in the study (110.0 cm ± 7.6 vs. 106.6 cm ± 7; p = 0.02). No significant differences were found in weight and a borderline significant difference was found in BMI z-scores (-0.18 ± 1.19 vs. 0.27 ± 0.96 (p = 0.05)).

Comparison of the study sample with a general population of families with 3-7 year old children living in Flanders in 2002 [15] revealed a possible selection bias in the study sample (Table 1) [15,16]. Children from lower educated parents were under-represented in our study sample. Also families who did not have an income from employment were under-represented in our study sample compared with the population sample.

**Table 1:** Characteristics of the children participating in the validity study in comparison with the Flemish population

	Belgian households with young children (3 - 7 y) (%)†	Families in the study (%) (n = 297)
Highest education of father & mothers*:		
Lower secondary education	19.4	11.3
Higher secondary education	42.1	34.9
Higher education (e.g. bachelor)	21.1	32.3
University degree (e.g. master degree)	17.5	21.5
Income status of families:		
Two incomes from employment	72.2	70.8
One incomes from employment	12.7	23.5
No income from employment (e.g. only replacement income(s))	15.1	5.8

* Distribution of educational levels of mothers and fathers in the Flemish population 25-34 years old in 2002 (The Flemish Authority, 2007)[16]

† Panel study of Belgian Households - year of observation 2002 was used as a reference (Child and Family, 2003)[15]

Children had a mean age of 4.8 years (SD ± 0.8 year) and an age range from 3.2 to 7.1 years (21% 3.2 to 3.9 y; 37% 4 to 4.9 y; 36% 5 to 5.9 y; 6% 6 to 7.1 y). Both sexes were almost equally represented in the study (47% girls). Seventy-four percent of the parents measured the weight of their child and 66% measured their child's height. Children were assigned to the following groups depending on whether their weight and/or height were estimated or measured by their parents:

- Height and weight estimated: 18.9% (n = 56)

- Height estimated and weight measured: 11.8% (n = 35)

- Weight estimated and height measured: 4.4% (n = 13)

- Height and Weight measured: 54.5% (n = 162)

In total, 263 (89%) of the questionnaires analyzed were answered by the mother of the child. No significant differences were found in socio-economic status between parents estimating their child's weight and/or height and those measuring their child's weight and height at home.

From table 2 it can be seen that parents slightly, but significantly, underestimated the weight of their child in comparison with the weight measured by the CLB nurse. They slightly overestimated the height of their child in comparison with the height measured by the CLB nurse, but this difference was not significant. This resulted in a significant underestimation of the BMI reported by the parents compared with the BMI calculated from the CLB data (Table 2). Mean differences between parent-reported and measured weight, height and BMI were larger when parents estimated their child's weight and height values than when they measured these parameters at home. However, t-tests for comparing 'differences of parent-report and index measurements' between the group of children with parent-estimates and those with parent-measurements, showed no significant differences between these two groups of children (p-values for weight, height and BMI were respectively 0.479, 0.812, and 0.961).

**Table 2:** Accuracy of parent-reported weight and height among preschool children: comparing parental measurements with parental estimates

Reporting method used by parents	Parent reported		Index Measured		Difference		P*	ICC	
	Mean (SD)		Mean (SD)		Mean (SD)			Intraclass correlation	95% CI
Weight (kg) (weight was estimated) (n = 71)	18.4	(3.8)	19.2	(3.7)	-0.75	(2.4)	0.009	0.793	(0.677-0.869)
Weight (kg) (weight was measured) (n = 204)	18.2	(3.4)	18.8	(3.5)	-0.60	(1.0)	< 0.001	0.942	(0.864-0.969)
Height (cm) (height was estimated) (n = 94)	109.0	(8.0)	108.7	(7.3)	+0.27	(4.7)	0.578	0.811	(0.728-0.870)
Height (cm) (height was measured) (n = 183)	109.2	(8.3)	109.0	(7.9)	+0.17	(2.5)	0.370	0.951	(0.935-0.963)
BMI (kg/m²) (weight and/or height were estimated) (n = 104)	15.3	(2.2)	15.9	(1.7)	-0.56	(2.0)	< 0.006	0.456	(0.288-0.596)
BMI (kg/m²) (weight and height were measured) (n = 162)	15.2	(1.4)	15.8	(1.5)	-0.55	(1.1)	< 0.001	0.671	(0.485-0.783)

* According to the Paired samples t-test

ICC: Intraclass correlation coefficient

For the three body dimensions (weight, height and BMI), much larger limits of agreement (mean difference ± 1.96SD) were found for the group of children whose weight and height were estimated by their parents in comparison with the children whose weight and height were measured at home.

The intraclass correlation coefficients between index measured and reported weight, height and BMI-values indicate that the associations were stronger when parents measured their children at home than when they estimated the weight and/or height values without measurements (see Table 2).

The proportions of children being categorized as underweight, overweight/obese and obese, based on international cut-off values [12], are presented in table 3. Using index measured weight and height values for BMI calculations, more children were identified as being overweight or obese than when parent-reported weight and height values were used. However, when using parent-reported weight and height values, the prevalence of underweight was significantly higher than when actual index weight and height values were used.

**Table 3:** Proportions of children categorized as underweight, overweight/obese and obese

Reporting method used by parents	Parent reported		Index Measured		Difference		P*
		
	%	(n)	%	(n)	%	(SD)	
Underweight (weight and/or height estimated) (n = 104)	27.9	(29)	10.6	(11)	17.3	(4.8)	< 0.001
Underweight (weight and height measured) (n = 162)	23.5	(38)	9.3	(15)	14.2	(3.3)	< 0.001
Overweight/obese (weight and/or height estimated) (n = 104)	13.5	(14)	16.3	(17)	-2.8	(3.7)	0.607
Overweight/obese (weight and height measured) (n = 162)	8.0	(13)	11.7	(19)	-3.7	(2.3)	0.180
Obese (weight and/or height estimated) (n = 104)	2.9	(3)	3.8	(4)	-0.9	(2.5)	1.000
Obese (weight and height measured) (n = 162)	1.2	(2)	2.5	(4)	-1.3	(1.2)	0.625

* According to the McNemar's test

Misclassification analysis indicated that more children were grossly misclassified when parents estimated their child's weight and height than when they measured these values at home, while fewer children were classified correctly (Table 4). The weighted kappa statistic for BMI-categories (4 categories) was higher in the group of parents who measured weight and height at home, in comparison with parents who estimated these parameters. Though, 95% confidence intervals were large and showed some overlap.

**Table 4:** Cross-classification analyses for parent-reported (measured versus estimated) and accurately measured (by school nurse) BMI-categories*

	Reported versus measured BMI				
			
Parental report	Same category (%)	Adjacent category (%)	Extreme category (%)	Weighted kappa (95% CI)	
Weight and height estimated	55	42	3	0.25	(0.07 to 0.43)
Weight and height measured at home	72	25	2	0.39	(0.22 to 0.57)

* The IOTF cut-off values for determining underweight, normal weight, overweight, and obesity in the 266 children

The validity tests for classifying underweight, overweight and obesity from the parent-reported weight and height, using the CLB measurements as a reference, are shown in table 5. The sensitivity for identifying the presence of underweight, overweight and obesity status, based on parent-reported BMI, compared with measured BMI, was lower when parents estimated their child's weight and height than when they measured these values at home. Also, specificity was lower when parents estimated their child's weight and height than when they measured these values at home. The kappa statistic shows that agreement for underweight, overweight and obesity between parent-reported and index measured values was always higher when parents measured their child at home than when they estimated their child's weight and height without measurement.

**Table 5:** Diagnostic values of parent-reported (measured versus estimated) height and weight in detection of BMI-categories*

	Sensitivity % (95% CI)		Specificity % (95% CI)		Kappa Statistic (95% CI)	
	
Reporting method used by parents	Estimated	Measured	Estimated	Measured	Estimated	Measured
Underweight	55 (0.28 to 0.79)	73 (0.48 to 0.89)	75 (0.66 to 0.83)	82 (0.75 to 0.87)	0.17 (-0.10 to 0.45)	0.33 (0.08 to 0.57)
Overweight/Obese	47 (0.26 to 0.69)	47 (0.27 to 0.68)	93 (0.86 to 0.97)	97 (0.93 to 0.99)	0.43 (0.10 to 0.76)	0.52 (0.19 to 0.85)

* The IOTF cut-off values for determining underweight, normal weight, overweight, and obesity in the 266 children

When comparing the group of parents who estimated their child's weight and height with those measuring their child's weight and height for underestimation and overestimation of the BMI with at least 1 kg/m^2^, it was found that the prevalence of BMI under- and overestimations in the group of parents who estimated weight and height was significantly higher than the prevalence in the group of parents who measured weight and height (Table 6).

**Table 6:** Under- and overestimation of BMI according to characteristics of children and their parents

	Proportion with difference in BMI < -1 kg/m², % (*n/N*)		P*	Proportion with difference in BMI > +1 kg/m², % (*n/N*)		P*
Parental report:						
Height estimated, weight estimated	50.0	(28/56)		19.6	(11/56)	
Height estimated, weight measured	31.4	(11/35)		17.1	(6/35)	
Height measured, weight estimated	46.2	(6/13)		7.7	(1/13)	
Height measured, weight measured	27.2	(44/162)	0.01	4.3	(7/162)	0.01

* according to Fisher's exact test

*n *= number of cases underestimating/overestimating BMI with > 1 kg/m^2 ^(e.g. difference in BMI less than -1 kg/m^2^)

*N *= total number of cases with the specified characteristic (e.g. number of boys; number of children < 5 years old; etc.)

## Discussion

### Principal findings

More than 60% of the parents measured their child's weight and height at home before registering these values in the questionnaire. This number might have been inflated because the option for parents to report measured values motivated them to measure their child's anthropometrics. Alternatively, some parents were likely not prompted to measure their child because they were confident of its accuracy because it was measured somewhere in the recent past.

In general, agreement between parent-reported and index measured weight and height values, accordingly BMI-values, was rather low for both parent measured and parent estimated anthropometrics. Only for overweight, was a moderate agreement found, while for underweight and obesity, only poor to fair agreements were found from the kappa statistics, indicating limited accuracy and utility of parent-reported weight and height.

For each of the 3 parameters (weight, height and BMI), the ICC correlations and weighted kappa statistics were higher in the group of children whose parents measured their body parameters at home, in comparison with the children whose parents estimated their weights or heights.

Much larger limits of agreement were found for the group of children whose weight and height were estimated by their parents in comparison with the children whose weight and height were measured at home. These larger limits of agreement suggest that the errors in weight, height and BMI are higher when parents are estimating their child's weight and height values than when they measure them at home. Thus, when using parent-reported data on an individual level, the accuracy may be improved by encouraging parents to measure the weight and height of their child at home.

Parent reports of a child' height and weight without measurement at home are interesting entities. Presumably, parents will have obtained this information from others who probably measured their child in other settings, e.g., schools, clinics, or paediatricians. The relative closeness of the parent estimates to the index measurements suggests that the information was obtained fairly recently. Interestingly, the parent estimations for height overestimated the index measured height by a small amount (mean difference 0.27 cm). This is interesting because any information regarding the child's height must have been obtained sometime before the parent completed the questionnaire. If much time elapsed since the information was obtained, one would think that parent estimates would be systematic underestimates of the index height because of subsequent height growth during the ensuing period. Not only time-gaps between the time of measurement and the time of completing the questionnaire could bias the parent estimates, but also the measurement method could possibly partly explain the differences found between parental estimates and measurements (e.g. some paediatricians are measuring children's height while laying down, while CLB-measures were obtained in standing position).

However, from our results, it appears that the bias in parent-reported weight and height results not only from just estimating, but also from inexact measuring at home. Parent reports of a child's height and weight based on home measurements appear desirable. Nevertheless, the exact protocols used by parents are obviously varied and really unknown in the present study because no measurement instructions were given. Therefore, future studies should examine whether a simple protocol/instructions for more standardized measuring weight and height of children at home could further improve the accuracy of these parent reports.

### Methodological issues and limitations

Some limitations of this study are worth noting. Data were available only for children whose parents completed the questionnaire. Children who were measured by a CLB nurse but whose parents did not complete the questionnaire were excluded from the analyses. It is possible that respondents were more willing, or more able, than non-respondents to provide accurate assessments of their children's weight and height. Therefore, the errors between parentally reported and measured weight and height in this sample may be underestimates of the true errors, since almost 30% of the parents refused to complete the questionnaire. However, to avoid underestimation of the true errors, the subjects were not aware of the future comparison between reported and measured values.

A more in depth comparison of some of the characteristics of the children and their parents with characteristics of a population of 'Flemish preschoolers' revealed that higher educated parents were overrepresented. Also families who do not have an income from employment (both mother and father are not employed) were underrepresented in our study sample in comparison with families with 3-7 years old children living in Flanders. Therefore, our study sample of children participating in this validation study seems to be subject to some selection bias, in which higher social classes are likely to be overrepresented. It is unknown whether these factors are related to differential parent reporting of child measurements.

Furthermore, in this study, the examination by the CLB nurses, during which weight and height were measured, was performed about 2 weeks after completion of the questionnaire. As there might be up to 2 weeks between the two assessments, the true weight and height might change during this period. However, large changes, which might influence the present results, are unlikely to have occurred during that period.

An important strength of this study is the high level of standardization in the reference measurements performed by the experienced and trained CLB nurses, and the inclusion of both parent-measured and parent-estimated child dimensions.

### Comparison with previous studies

Studies concerning the validity of parent-reported weight and height in preschool children are scarce [10]. Few studies examined the validity of parent-reported weight and height of their child, though the results are rather difficult to compare due to different study protocols or analyses and disparate child ages [7,17-19]. Therefore, it is difficult to generalize the results derived from these studies [10]. To our knowledge, this is the first study to investigate differences in accuracy of weight, height and BMI values reported by parents, based on estimated compared to parental measurements of the child at home. Bradley et al. estimated the validity of parental measurements of infant size, using illustrated instructions and simple measuring tools, and they found that their tested methods were suitable for ranking individuals and for use in group-level analyses [20]. Therefore, it may be that a similar approach in preschool children could also improve the validity of parent reports. As mentioned before, future research should investigate whether a simple protocol for measuring weight and height of the child at home could further improve the accuracy of parental reports in large-scale surveys.

## Conclusions

In conclusion, our results demonstrate the degree of inaccuracy of parent-reported weight and height values in classifying preschool children as being underweight, overweight or obese. However, the important differences found between parent-measured weight and height values compared with parent-estimated values, suggest the importance of motivating the parents to measure their child at home when the study design includes the use of parent reports for weight and height values of their children. Nevertheless, future research should investigate the degree to which appropriate illustrated instructions could further improve the accuracy of such parent reports.

## Abbreviations

CLB: Centre for Pupils Counseling (Centrum voor Leerlingenbegeleiding in Dutch); BMI: Body Mass Index; WHO: World Health Organization; SPSS: Statistical Package for the Social Sciences; Yrs: years; SD: Standard Deviation; CI: Confidence Interval; ICC: Interclass Correlation Coefficient

## Competing interests

The authors declare that they have no competing interests.

## Authors' contributions

The original idea for the analyses came from JHH. IH did all of the data management and analysis under the supervision of SDH and DDB as part of her PhD degree at the Ghent University. IH led on the writing of the paper but JHH contributed importantly to the different drafts of the paper and suggested analysis for the manuscript. IVT supervised the local fieldworkers and BC assisted in the analyses. CO, TDV, JHH, DDB, WDK and BC also assisted in the interpretation of the analyses and in part of the data cleaning. All authors have read the final version of the manuscript before submission.

## Pre-publication history

The pre-publication history for this paper can be accessed here:

http://www.biomedcentral.com/1471-2431/11/63/prepub
